# Extracellular vesicles derived from glioblastoma promote proliferation and migration of neural progenitor cells via PI3K-Akt pathway

**DOI:** 10.1186/s12964-021-00760-9

**Published:** 2022-01-12

**Authors:** Jiabin Pan, Shiyang Sheng, Ling Ye, Xiaonan Xu, Yizhao Ma, Xuanran Feng, Lisha Qiu, Zhaohuan Fan, Yi Wang, Xiaohuan Xia, Jialin C. Zheng

**Affiliations:** 1 Center for Translational Neurodegeneration and Regenerative Therapy, Shanghai Tenth People’s Hospital Affiliated to Tongji University School of Medicine, Shanghai, 200072 China; 2 Translational Research Institute of Brain and Brain-Like Intelligence, Shanghai Fourth People’s Hospital Affiliated to Tongji University School of Medicine, Shanghai, 200434 China; 3 Collaborative Innovation Center for Brain Science, Tongji University, Shanghai, 200092 China

**Keywords:** Glioblastoma, Extracellular vesicles, Neural progenitor cells, Proliferation, Migration, PI3K, Akt

## Abstract

**Background:**

Glioblastomas are lethal brain tumors under the current combinatorial therapeutic strategy that includes surgery, chemo- and radio-therapies. Extensive changes in the tumor microenvironment is a key reason for resistance to chemo- or radio-therapy and frequent tumor recurrences. Understanding the tumor-nontumor cell interaction in TME is critical for developing new therapy. Glioblastomas are known to recruit normal cells in their environs to sustain growth and encroachment into other regions. Neural progenitor cells (NPCs) have been noted to migrate towards the site of glioblastomas, however, the detailed mechanisms underlying glioblastoma-mediated NPCs’ alteration remain unkown.

**Methods:**

We collected EVs in the culture medium of three classic glioblastoma cell lines, U87 and A172 (male cell lines), and LN229 (female cell line). U87, A172, and LN229 were co-cultured with their corresponding EVs, respectively. Mouse NPCs (mNPCs) were co-cultured with glioblastoma-derived EVs. The proliferation and migration of tumor cells and mNPCs after EVs treatment were examined. Proteomic analysis and western blotting were utilized to identify the underlying mechanisms of glioblastoma-derived EVs-induced alterations in mNPCs.

**Results:**

We first show that glioblastoma cell lines U87-, A172-, and LN229-derived EVs were essential for glioblastoma cell prolifeartion and migration. We then demonstrated that glioblastoma-derived EVs dramatically promoted NPC proliferation and migration. Mechanistic studies identify that glioblastoma-derived EVs achieve their functions via activating PI3K-Akt-mTOR pathway in mNPCs. Inhibiting PI3K-Akt pathway reversed the elevated prolfieration and migration of glioblastoma-derived EVs-treated mNPCs.

**Conclusion:**

Our findings demonstrate that EVs play a key role in intercellular communication in tumor microenvironment. Inhibition of the tumorgenic EVs-mediated PI3K-Akt-mTOR pathway activation might be a novel strategy to shed light on glioblastoma therapy.

**Video Abstract**

**Supplementary Information:**

The online version contains supplementary material available at 10.1186/s12964-021-00760-9.

## Background

Glioblastomas are the most malignant gliomas (grade IV glioma) that contain a large proportion of dividing tumor cells nourished by ample and abnormal blood supply [1, 2]. They are highly infiltrative and invade other brain regions. Although surgery, radiation, and chemotherapy are developed for treating glioblastoma, no cure is available and the standard care for glioblastoma remains almost unchanged for nearly 20 years with a median life expectancy of 15 months from the time of diagnosis to death [3]. Given that, new insights on glioblastoma tumor cells and their surrounding cells are in urgent need for the development of new therapy.

Invasive growth of glioblastomas that depletes a thorough tumor resection and extensive changes in the tumor microenvironment (TME) are main reasons for observed resistance to current therapy and concomitant recurrence [4–6]. Glioblastomas are known to have the capacity to recruit and alter the phenotypes of normal cells in their environs to sustain growth and encroachment into other regions [7]. Numerous forms of cell–cell communication are utilized by glioblastomas to “hijack” cells in the TME to promote tumor progression [3–5]. These routes include direct secretion of soluble factors, exchange of proteins and molecules via gap junctions, and transfer cellular contents via extracellular vesicles (EVs) [8]. Through these routes, glioblastomas alter immune cell function, sustain tumor vasculature, cause neurotoxicity, and alter astroglia phenotypes. Formidably, a dominant proportion of surrounding cells further provide feedback to potentiate glioblastoma aggressiveness via this crosstalk [9, 10].

Neural progenitor cells (NPCs) have been noted to migrate towards the site of glioblastomas in vitro under the influence of chemoattractant secreted by glioblastoma tumor cells [11, 12]. However, the specific mechanism of communication between glioblastoma cells and neural progenitor cells remains unknown. EVs are small lipid bilayer vesicles budding from cell plasm [13–15] or budding off the tips of nanotubes [16] that play as essential intercellular communicators [17]. Our previous work demonstrated that EVs retain certain properties of their donors and are able to confer donor properties to recipient cells [18]. Therefore, we hypothesized that glioblastoma-derived EVs may retain the tumor properties that altering the phenotype and function of surrounding NPCs. To test this premise, we utilized three classic glioblastoma cell lines, U87, A172, and LN229, and collected EVs in the culture medium of the three lines. Our results suggested that EVs derived from all three lines significantly promoted proliferation and migration of NPCs. Next, proteomics and western blotting analyses identified that PI3K-Akt pathway was enriched in glioblastoma-derived EVs and activated in EVs recipient cells in the meantime. The positive effects of glioblastoma-derived EVs on proliferation and migration of NPCs were further abrogated when recipient cells were treated with Wortmannin, a PI3K inhibitor [19], which, confirmed PI3K-Akt pathway as an essential downstream factor of aforementioned EVs. Thus, our findings revealed a novel mechanism for the glioma-mediated TME establishment that may shed light on the development of therapeutic strategy for treating glioblastoma with higher efficacy.

## Material and methods

### Animals and regents

C57BL/6J mice were purchased from Shanghai Laboratory Animal Center (Chinese Academy of Sciences) and were housed and maintained in the Comparative Medicine Facility of the Tongji University School of Medicine (Shanghai, China). All procedures were conducted in accordance with the protocols approved by the Institutional Animal Care and Use Committee at Tongji University School of Medicine.

### Mouse NPCs (mNPCs) culture and treatment

Mouse cortical NPCs were isolated from gestational day E14 brain tissue as previously described [20]. Brain tissues were dissected and mechanically dissociated using forceps to remove the membranes and large blood vessels. Brain tissues were digested by Trypsin–EDTA (Life Technologies) and then plated on cell culture flasks in mouse NeuroCult NSC Proliferation Medium (StemCell Technologies, Vancouver, BC, Canada), supplemented with epidermal growth factor (EGF, 10 ng/ml, Novus Biologicals), basic fibroblast growth factor (bFGF, 20 ng/ml, Novus Biologicals) for selective neurosphere cultures and penicillin/streptomycin (1% v/v, Gibco). Neurospheres were passaged when they reached 100–150 μm in diameter.

### U87 and A172 culture

The human glioblastoma cell lines U87 and A172 (American Type Culture Collection, Manassas, VA, USA) were authenticated by American Type Culture Collection using the short tandem repeat genotyping method. U87 and A172 cells were cultured in DMEM GlutaMax (Gibco) containing 10% FBS (Sigma-Aldrich) and penicillin/streptomycin (1% v/v) in humidified chamber (37 °C, 5% CO_2_ incubator). U87 cells were passaged at 3–4 day intervals.

### Isolation of EVs

The method for the isolation of extracellular vesicles has been described previously [21]. Briefly, 1 × 10^6^ U87 or A172 cells were plated on 10 cm dish and grown to 70–80% confluence. Then, U87s or A172 were rinsed with PBS three times and incubated with serum-free DMEM GluMax for 24 h. Media were harvested and first centrifuged at 300×*g* for 10 min to remove free cells, at 3,000×*g* for 20 min to remove cellular debris, and then at 10,000×*g* for 30 min to remove intracellular organelles. Lastly, EVs were collected by ultracentrifugation at 100,000×*g* for 2 h. All centrifugation steps were performed at 4 °C.

### Nanoparticle tracking analysis (NTA)

The size and concentration of extracellular vesicles were measured with NanoSight NS300 system (Malvern Instruments, UK). Briefly, U87 and A172 cells were cultured in 10 cm culture dishes for 48 h. Then, the medium was changed to serum-free medium for 24 h. The supernatants were differentially centrifugated and resuspended with 100 μl PBS and diluted at 1:10 in PBS, and then 1 ml solution was used for NTA analysis.

### Western blotting

EV pellets or cells were lysed in M-PER mammalian protein extraction reagent (Thermo Scientific) containing protease inhibitor (Thermo Scientific). Protein concentration was determined using the BCA (bicinchoninic acid) Protein Assay Kit (Pierce). An analytical 10% SDS polyacrylamide gel electrophoresis (SDS PAGE) was prepared and then transferred to polyvinyldifluoridene (PVDF) membranes (Millipore, Billerica, MA, USA). After blocking in 5% fat-free milk for 1 h, the membrane was incubated with purified primary antibodies for phospho-PI3K (p-PI3K, 1:1,000; Cell Signaling Technologies), PI3K (1:1,000; Cell Signaling Technologies), phospho-Akt (Ser473) (p-Akt, 1:1,000; Cell Signaling Technologies), Akt (1:1,000; Cell Signaling Technologies), phospho-mTOR (p-mTOR, 1:1,000; Cell Signaling Technologies), mTOR (1:1,000; Cell Signaling Technologies), phospho-C-Raf (p–C-Raf, 1:1000; Cell Signaling Technologies), phospho-MEK1/2 (p-MEK1/2, 1:1,000; Cell Signaling Technologies), MEK1/2 (1:1,000; Cell Signaling Technologies), phospho-ERK1/2 (p-ERK1/2, 1:1,000; Cell Signaling Technologies), ERK1/2 (1:1,000; Cell Signaling Technologies), β-actin (Actin, 1:5,000; Proteintech), flotillin-1 (1:1,000; BDbiosciences), flotillin-2 (1:5,000; BDbiosciences), TSG101 (1:5,000; Abacm), Alix (1:2,000; Bioworld), Calreticulin (1:1,000; Abcam) overnight at 4 °C followed by a horseradish peroxidase-linked secondary anti-rabbit or anti-mouse antibody (1:5,000; Icllab). Antigen–antibody complexes were visualized by Pierce ECL Western Blotting Substrate (Thermo Scientific).

### Immunocytochemistry

For immunofluorescence staining, mNPCs were plated on the coverslips and fixed using 4% paraformaldehyde (PFA) for 20 min, then permeabilized with 0.4% Triton-X in PBS for 15 min. Subsequently, the coverslips were blocked with1% BSA for 1 h. mNPCs were incubated with primary antibodies overnight including Nestin (1:500; Novus Biologicals) and Ki67 (1:1,000; Cell Signaling Technologies). Coverslips were washed with PBS 3 times and incubated for 1 h at room temperature with secondary antibodies including anti-rabbit, mouse or chicken IgG (coupled with Alexa Fluor 488 or 568, Life Technologies). Nuclei were counter-stained with DAPI. Coverglasses were fixed on glass slides with Mounting Medium (Sigma-Aldrich). The images were captured by Zeiss AX10 fluorescence microscope. For quantification, the numbers of stained cells were quantified by Image-Pro Plus 6.0.

### Transwell assay

8 mm pore size transwell system (Costar) were coated with diluted matrigel (1:80, matrigel:NPC basal medium) in humidified chamber (37 °C, 5% CO_2_ incubator) for 30 min. Briefly, NPCs were dissociated into single cells and 2 × 10^5^ cells/ml were resuspended in Mouse NeuroCult Proliferation Medium. The top chamber of the transwell was loaded with 100 μl of cell suspension containing either EVs or PBS. In the lower chamber, 600 μl of NPC proliferation medium was added. After 12 h, the transwell inserts member was fixed with 4% PFA, and cells were removed by a cotton swab from the upper chamber. Migrated cells on the bottom of the membrane were stained with DAPI (Sigma Aldrich). For each insert, cells migrated through the pores were captured by Zeiss AX10 fluorescence microscope. Cell numbers were counted using Image-Pro Plus 6.0. The cell number of each insert-treated group was normalized to the cell number of the control group to analyze migration index.

### Wound healing assay

mNPCs were seeded at 80% confluence in a 24-well plate coated with diluted matrigel. Each well was scratched using a 200 µl pipette tip. Each well was washed with PBS for three times and added NPC proliferation medium containing EVs or PBS. Images were captured at 0 h and 24 h after the initial scratch. Images were captured by Olympus light microscopy.

### EdU incorporation assay

Click-iT® EdU Imaging Kits (Thermo Scientific, #C10338) was used to analyze DNA synthesis according to the manufacturer’s instructions. 2.5 × 10^5^ mNPCs cell were planted on 35 mm Coverglass-Bottom Dish (Cellvis, #D35-14-1-N). After 24 h, medium was changed with fresh medium containing 50 μg/ml EVs or PBS for 24 h. EdU was added to medium before 2 h of fixation. Then, cells were fixed using 4% PFA for 20 min, and permeabilized with 0.5% triton-X100 in PBS for 15 min. Click-iT® reaction cocktails (Thermo Scientific) were added to dish for reacting for 30 min at room temperature and protected from light. Subsequently, cell nuclei were stained with DAPI. For each dish, 6 fields were randomly taken using Zeiss AX10 fluorescence microscope. The numbers of EdU-labled and DAPI-stained cells were counted by Image-Pro Plus 6.0.

### Cell proliferation assay

Briefly, 15 μg/ml glioblastoma-derived EVs were co-cultured with 5000 cells/well mNPCs on 96-well plates for 24 h, then changed with NPC proliferation medium without EVs and cultured for 48 h. Cell viability was measured by CCK-8 (Yeasen, #40203ES80) assays at different time points. Experiments were handled according to the manufacturer's instructions. Absorbance was measured at 570 nm and 450 nm and analyzed using SpectraMax M5 microplate readers (Molecular Devices).

### Protein identification and bioinformatics analysis

The resulting MS/MS data were processed using Maxquant search engine (v.1.5.2.8). Tandem mass spectra were searched against uniprot database concatenated with reverse decoy database. Trypsin/P was specified as cleavage enzyme allowing up to 4 missing cleavages. FDR was adjusted to < 1% and minimum score for modified peptides was set > 40. Proteins were classified by Gene Ontology (GO) annotation into three categories: biological process, cellular compartment, and molecular function. For each category, a two-tailed Fisher’s exact test was employed to test the enrichment of the differentially expressed protein against all identified proteins. The GO with a corrected *p*-value < 0.05 is considered significant. Encyclopedia of Genes and Genomes (KEGG) database was used to identify enriched pathways by a two-tailed Fisher’s exact test to test the enrichment of the differentially expressed protein against all identified proteins. The pathway with a corrected *p*-value < 0.05 was considered significant.

### Enzyme-linked immunosorbent assay (ELISA)

Cultured medium of NPCs with/without EVs treatment was collected. The concentration of pro-inflammatory cytokine tumor necrosis factor alpha (TNF-α) and interleukin (IL)-1β in the culture medium was measured with commercially available ELISA kits (Mouse TNF alpha ELISA Kit cat# abs520010, absin; Mouse IL-1β/IL-1F2 Immunoassay, cat# MLB00C, R&D) according to manufacturer’s protocols.

### Quantitative reverse transcription-polymerase chain reaction (qRT-PCR)

The mRNA was extracted from EVs-treated mNPCs using RNeasy mini kit (Qiagen) according to the manufacturer’s instructions. Genomic DNA was removed using DNase I digestion kit (Qiagen). cDNA was synthesized using miScript II reverse transcription kit (Qiagen). Transcripts were amplified using gene-specific primer (*Gpadh* forward primer: 5′-CATGTTCCAGTATGACTCCACTC-3′, *Gapdh* reverse primer: 5′-GGCCTCACCCCATTTGATGT-3′; *βIII-tubulin* forward primer: 5′-CTTTATCTTCGGTCAGAGTGGTGC-3′, *βIII-tubulin* reverse primer: 5′-TTCTTTCCGCACGACATCTAGG-3′; *Gfap* forward primer: 5′-TTGCTGGAGGGCGAAGAAAA-3′, *Gfap* reverse primer: 5′-CATCCCGCATCTCCACAGTC-3′) and SYBR green PCR kit (Qiagen) with the ABI7500 (Applied Biosystems). All qRT-PCR results measured each sample in triplicate and no-template blanks were used for negative controls. Amplification curves and gene expression were normalized to the house-keeping gene *Gapdh*.

### Statistical analyses

All results are the means of at least three independent experiments ± SD. Data from two groups were evaluated statistically by two-tailed, paired or unpaired student *t* test, and that among more than two groups was assessed with the parametric one-way ANOVA with post-hoc Bonferroni test. *p < 0.05, **p < 0.01, ***p < 0.001 and ****p < 0.0001, in comparison to control.

## Results

### Characterization of EVs released from U87, A172, and LN229 cells

We firstly characterized U87-derived EVs (U87-EVs), A172-derived EVs (A172-EVs), and LN229-derived EVs (LN229-EVs). EVs were collected using differential centrifugation from the serum-free culture media of U87, A172, and LN229 cells. NTA was utilized to analyze the size of EVs. NTA analyses demonstrated similar sizes for EVs released from U87 (Additional file 1: Fig. S1A), A172 (Additional file 1: Fig. S1B), and LN229 (Additional file 1: Fig. S1C), suggesting there was no significant difference in size distributions of different types of EVs. We then applied cells lysis, U87-EVs, A172-EVs, and LN229-EVs to Western blotting to confirm the expression of specific EVs markers Alix (ALG-2 interacting protein), Flotillin-1, Flotllin-2 (Additional file 1: Fig. S1D-F). The absence of lipoprotein marker ApoA1 and endoplastimc reticulum marker Calnexin in EVs indicates the purification of EVs without contamination of cell debris and organelles (Additional file 1: Fig. S1D-F).

### EVs released from glioblastoma increase glioblastoma cell proliferation

**Fig. 1:**
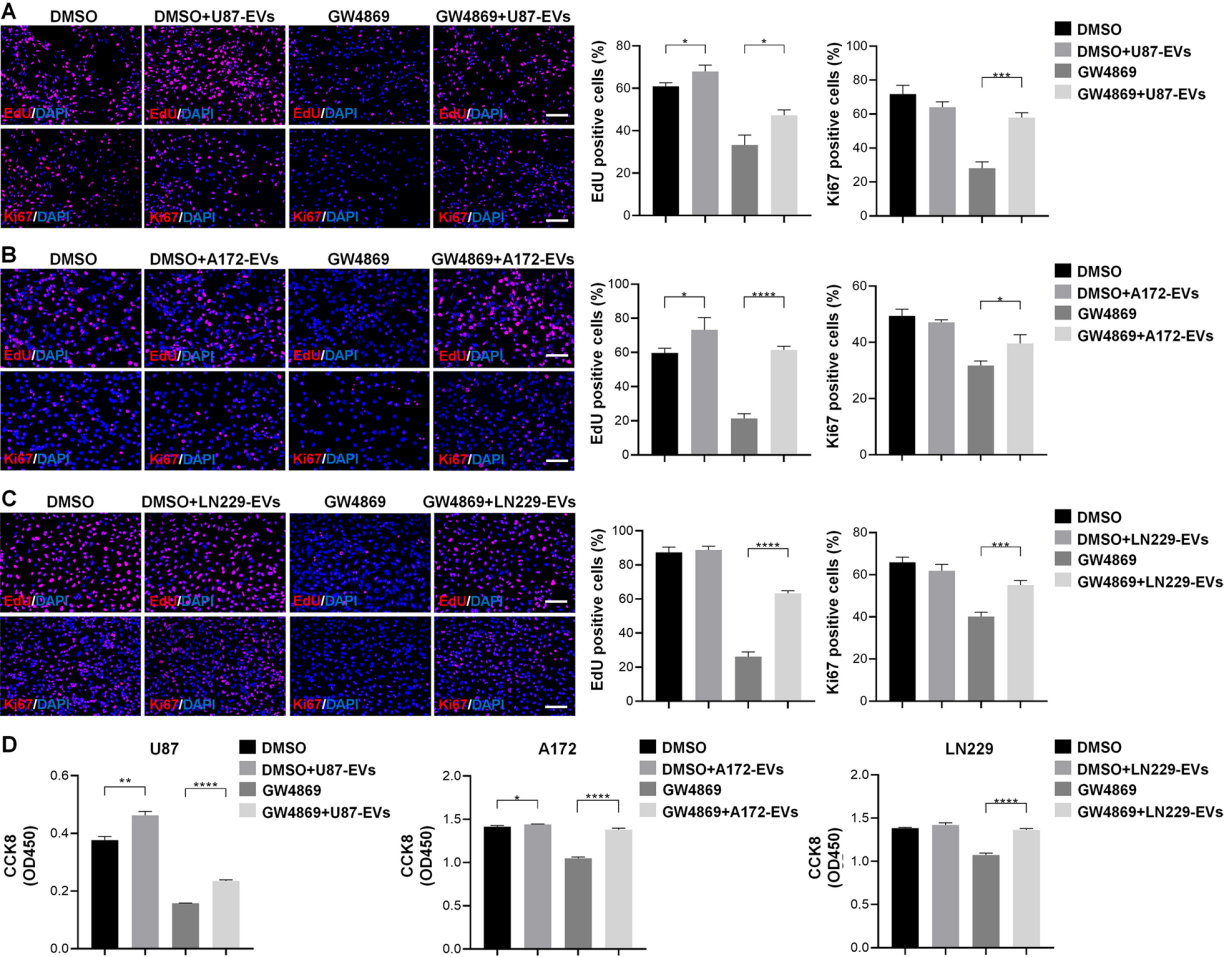
Glioblastoma-derived EVs promote the proliferation of glioblastoma cells. **A.** U87-EVs-treated U87 cells exhibited higher proportion of EdU positive cells than DMSO-treated cells in the EdU incorporation assay. GW4869 lowered the proportions of EdU positive signal in U87 cells. The addition of U87-derived EVs partially reversed the GW4869-reduced EdU positive signals. U87-EVs did not alter the proportions of Ki67 positive cells. GW4869 reduced the proportions of Ki67 positive cells, and the addition of U87-EVs partially reversed this reduction. **B.** A172-EVs-treated A172 cells exhibited higher proportion of EdU positive cells than DMSO-treated cells in the EdU incorporation assay. GW4869 lowered the proportions of EdU positive signal in A172 cells. The addition of A172-derived EVs partially reversed the GW4869-reduced EdU positive signals. A172-EVs did not alter the proportions of Ki67 positive cells. GW4869 reduced the proportions of Ki67 positive cells, and the addition of A172-EVs partially reversed this reduction. **C.** LN229-EVs-treated LN229 cells exhibited higher proportion of EdU positive cells than DMSO-treated cells in the EdU incorporation assay. GW4869 lowered the proportions of EdU positive signal in LN229 cells. The addition of LN229-derived EVs partially reversed the GW4869-reduced EdU positive signals. LN229-EVs did not alter the proportions of Ki67 positive cells. GW4869 reduced the proportions of Ki67 positive cells, and the addition of LN229-EVs partially reversed this reduction. **D.** U87-EVs-treated U87 cells exhibited higher CCK8 signals. GW4869 largely lowered CCK8 signals and U87-EVs significantly reversed the CCK8 signal reduced by GW4869. A172-EVs-treated A172 cells exhibited higher CCK8 signals. GW4869 largely lowered CCK8 signals and A172-EVs significantly reversed the CCK8 signal reduced by GW4869. LN229-EVs did not alter CCK8 signals in LN229 cells. GW4869 largely lowered CCK8 signals and U87-EVs significantly reversed the CCK8 signal reduced by GW4869. Results are presented as the mean ± SD of three independent experiments. *p < 0.05, **p < 0.01, ***p < 0.001 and ****p < 0.0001. Scale bar, 100 μm

We hypothesized that glioblastoma-derived EVs trigger glioblastoma cell prolifeartion. To verify this premise, we examined the proliferation of U87 cells, A172 cells, and LN229 cells treated accordingly with U87-EVs, A172-EVs, and LN229-EVs with EdU or Ki67 assays. U87-EVs-treated U87 cells exhibited higher proportions of EdU positive cells than vehicle-treated U87 cells (Fig. 1A). GW4869 largely reduced the proportions of EdU positive cells, and the addition of EVs to GW4869-treated U87 cells rescued the reduced proportion of EdU positive cells. These results demonstrate the necessity of EVs in cellular proliferation. (Fig. 1A). EVs treatment did not alter the proportions of Ki67 positive U87 cells, however, GW4869 significantly lowered Ki67 signaling, confirming the necessity of EVs in U87 cellular proliferation. Addition of U87-derived EVs rescued GW4869-lowered proportions of Ki67 positive cells (Fig. 1A). Similar effects of EVs on glioblastoma cellular proliferation were observed in A172 cells (Fig. 1B) and LN229 cells (Fig. 1C) as demonstrated by EdU assay and Ki67 immune cytochemistry. Likewise, the effects of glioblastoma-derived EVs on glioglastoma cellular proliferation were confirmed by CCK8 assay in U87, A182, and LN229 cells (Fig. 1D).

### EVs released from glioblastoma increase glioblastoma cell migration

**Fig. 2:**
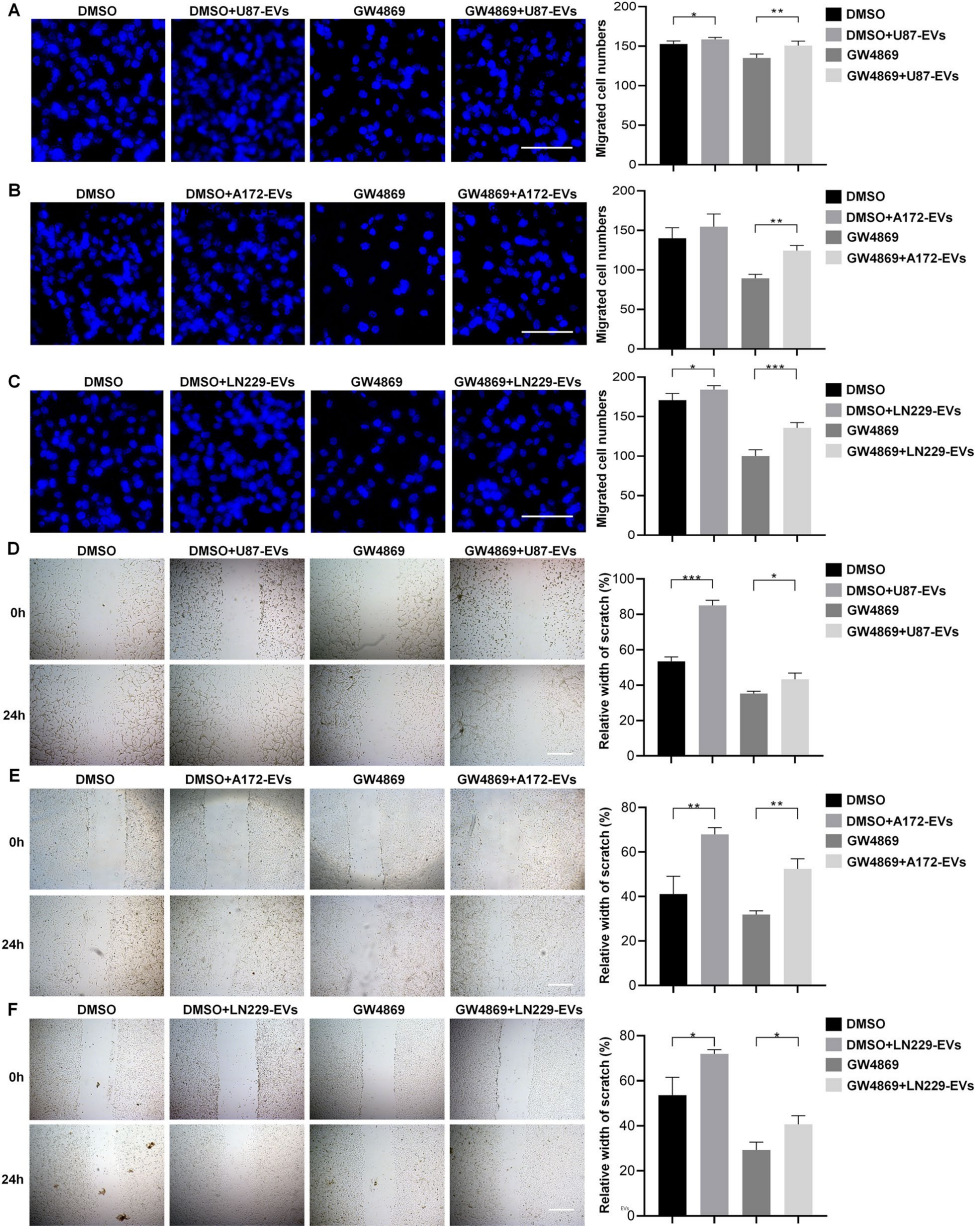
Glioblastoma-derived EVs elevate the migration of glioblastoma cells. **A** U87-EVs-treated U87 cells showed higher numbers of migrated cells than DMSO-treated cells in transwell assay. GW4869 largely reduced the number of migrated cells. The addition of U87-EVs to GW4869-treated cells significantly reversed GW4869-induced reduction of migrated cells. **B** A172-EVs did not alter the numbers of migrated A172 cells in transwell assay. GW4869 largely reduced the number of migrated cells. The addition of A172-EVs to GW4869-treated cells significantly reversed GW4869-induced reduction of migrated cells. **C** LN229-EVs-treated LN229 cells showed higher numbers of migrated cells than DMSO-treated cells in transwell assay. GW4869 largely reduced the number of migrated cells. The addition of LN229-EVs to GW4869-treated cells significantly reversed GW4869-induced reduction of migrated cells. Results are presented as the mean ± SD of three independent experiments. *p < 0.05, **p < 0.01, ***p < 0.001 and ****p < 0.0001. Scale bar, 100 μm (**A, B, C**) and 200 μm (**D, E, F**)

We utilized transwell assay and wound healing assay to investigate whether the EVs released from glioblastoma altered glioblastoma cell migration. In transwell assay, we found that the migrated cell number of U87 cells was slightly but significantly increased by U87-EVs. GW4869 treatment largely reduced migrated U87 cells, and the addition of U87-EVs significantly rescued GW4869-induced reduction in migrated cell number (Fig. 2A). These results indicated the necesssity of EVs in glioglatoma cell migration. Similar results were obtained from A172 cells and A172-EVs (Fig. 2B), as well as LN229 cells and LN229-EVs (Fig. 2C). In wound healing assay, our data showed that U87-EVs significantly promoted migratory capacity of U87 cells compared with DMSO-treated U87 cells. GW4869 largely lowered the migratory capacity of U87 cells, and the addition of U87-EVs to the culture system rescued ththe lowered migration (Fig. 2D). Similar results were obtained from A172 cells and A172-EVs (Fig. 2E), as well as LN229 cells and LN229-EVs (Fig. 2F). These results demonstrated that glioblastoma-derived EVs were critical for glioblastoma cell migration.

### EVs released from glioblastoma facilitate mNPC proliferation

**Fig. 3:**
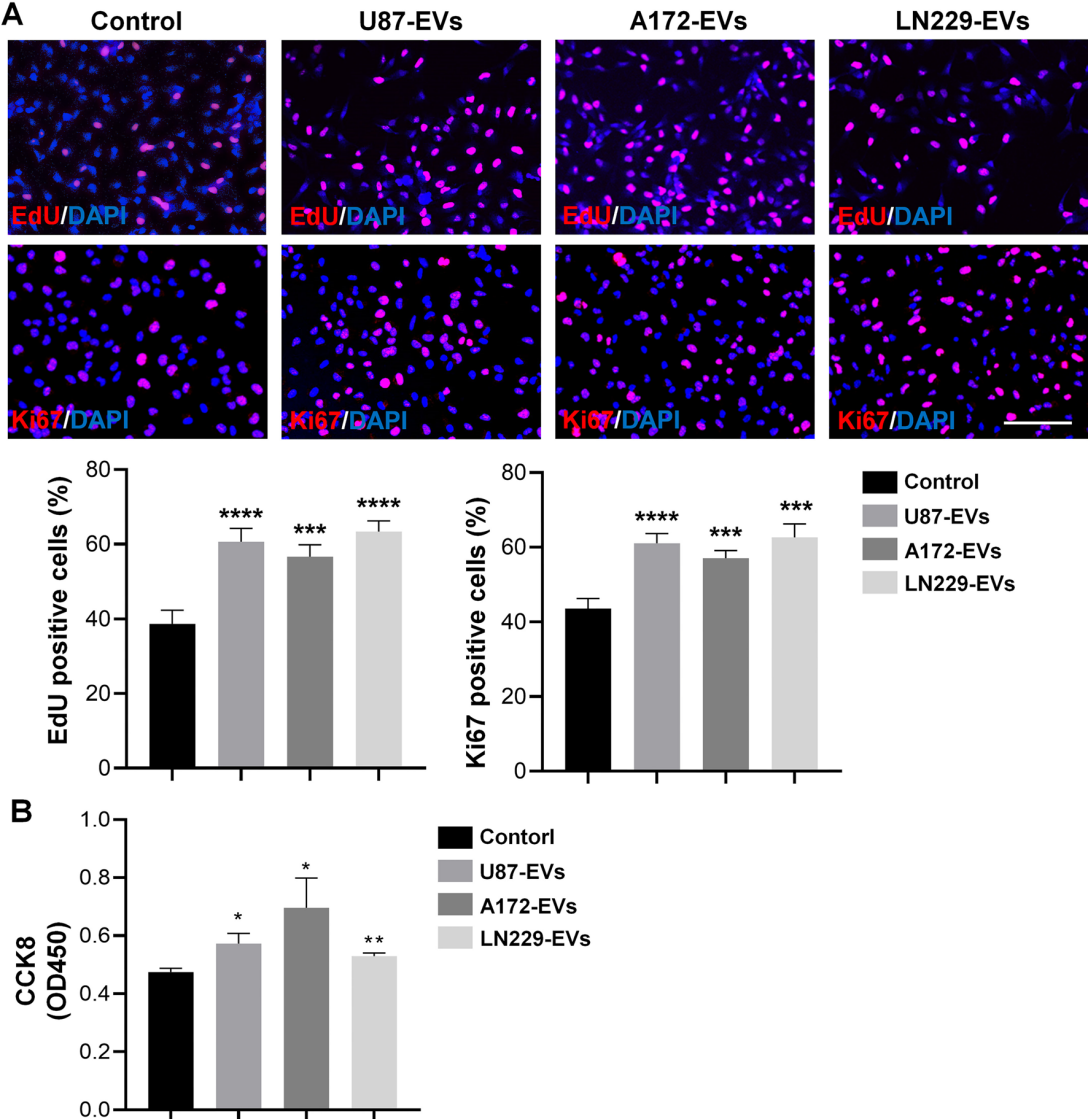
Glioblastoma-derived EVs promote the proliferation of mNPCs. **A** U87-EVs, A172-EVs, and LN229-EVs treatment all significantly increased the proportions of EdU positive or Ki67 positive mNPCs. **B** U87-EVs, A172-EVs, and LN229-EVs treatment all significantly increased CCK8 signals in mNPCs. *p < 0.05, **p < 0.01, ***p < 0.001 and ****p < 0.0001. Scale bar, 100 μm

Increasing evidences have shown abnormal proliferation of NPCs in glioblastoma microenvironment [21, 22]. We hypothesized that glioblastoma-derived EVs trigger NPCs’ proliferation in the patients with recurrent glioblastoma. To verify this premise, we examined the proliferation of mNPCs treated with either U87-EVs, A172-EVs, or LN229-EVs with EdU, Ki67, and CCK8 assays. U87-EVs, A172-EVs, and LN229-EVs treatment all increased the proportions of EdU positive cells and Ki67 positive cells in mNPCs (Fig. 3A). Likewise, U87-EVs, A172-EVs, and LN229-EVs treatment all increased CCK8 signals in mNPCs (Fig. 3B). These data indicated that glioblastoma-derived EVs facilitate mNPC proliferation.

### Glioblastoma-derived EVs enhance mNPC migration

**Fig. 4:**
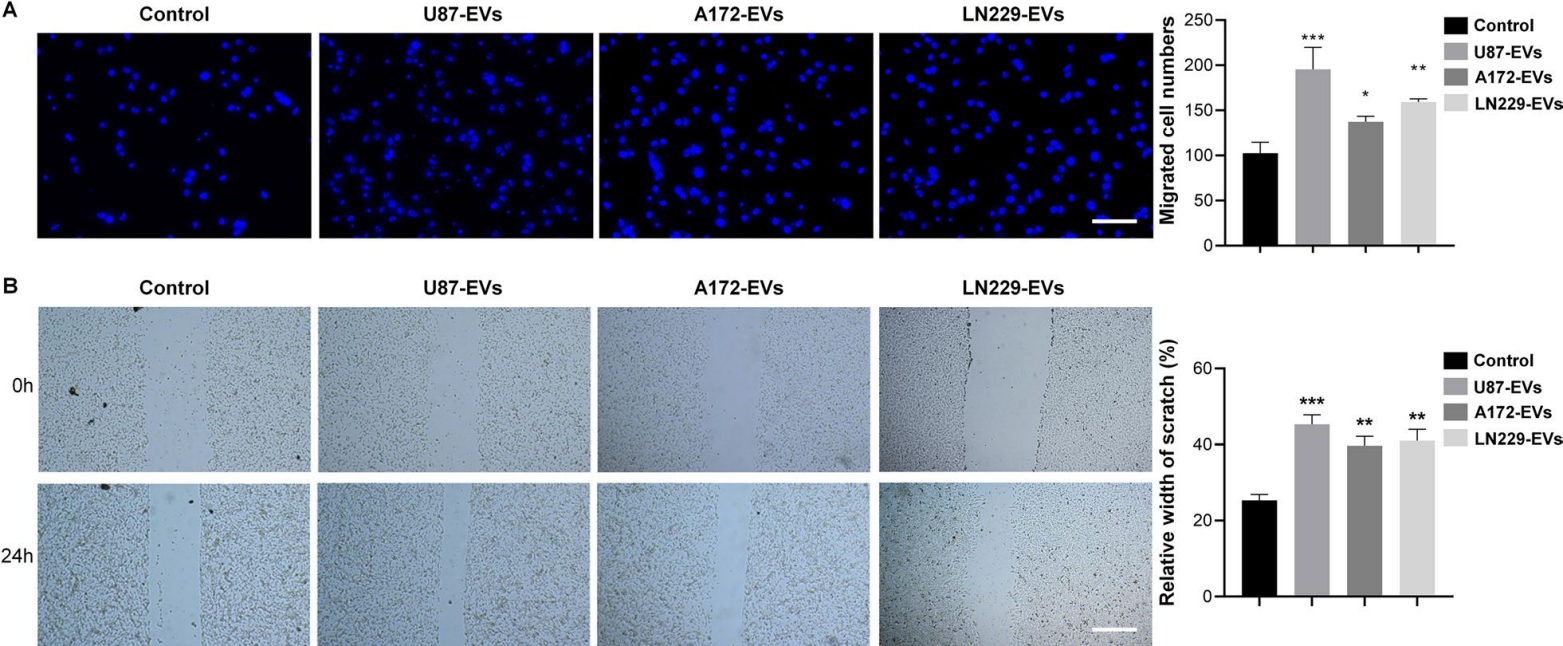
Glioblastoma-derived EVs elevate the migration of mNPCs. **A.** U87-EVs-, A172-EVs- and LN229-EVs-treated mNPCs all had significantly more migrated cells than control mNPCs in transwell assay. **B.** U87-EVs-, A172-EVs- and LN229-EVs-treated mNPCs showed significantly higher relative width of scratch than control mNPCs in wound-healing assay. Results are presented as the mean ± SD of three independent experiments. *p < 0.05, **p < 0.01, and ***p < 0.001, in comparison with the controls. Scale bar, 100 μm (**A**) and 200 μm (**B**)

Previous reports indicated that NPCs had a tropism for glioblastoma as NPCs implanted at distant sites away from the site of glioma migrated to the tumor across normal tissues [11, 12]. We utilized transwell assay and wound healing assay to investigate whether the EVs released from glioblastoma enhanced NPCs migration. In transwell assay, we found that the migration index of mNPCs was significantly enhanced by U87-EVs, A172-EVs, and LN229-EVs, compared with control mNPCs (Fig. 4A). In wound healing assay, our data also showed that U87-EVs, A172 EVs, and LN229-EVs all significantly promoted migratory capacity of mNPCs, compared with the controls (Fig. 4B).

### Glioblastoma-derived EVs altered cell signaling of mNPCs

**Fig. 5:**
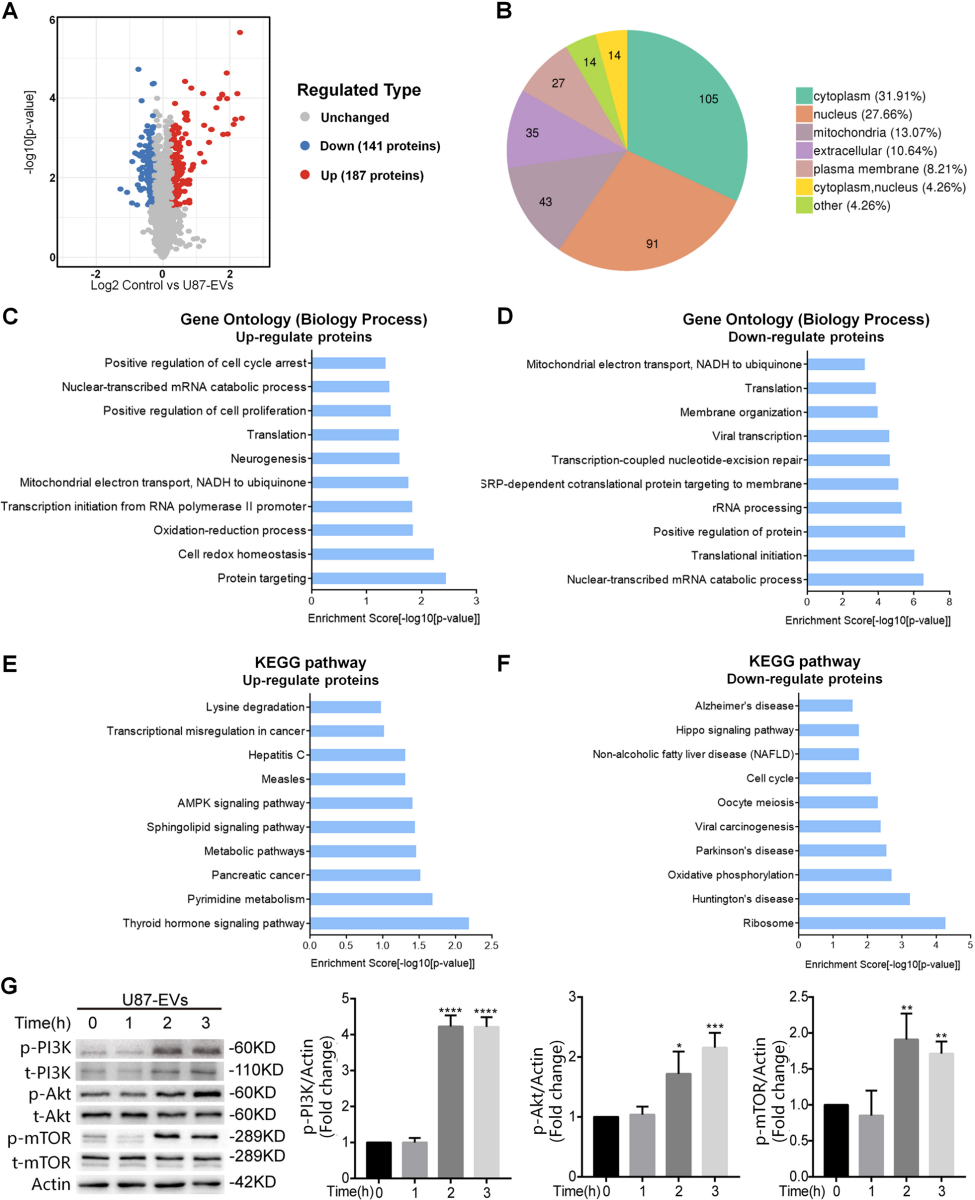
Proteomic analysis of U87 EVs-treated mNPCs revealed the activation of PI3K-Akt-mTOR pathway. **A** Proteomic analysis revealed 187 upregulated proteins and 147 downregulated proteins in U87-EVs-treated mNPCs compared with control mNPCs. **B** The distribution of changed proteins in U87-EVs-treated mNPCs. **C** Top 10 biology processes of upregulated proteins revealed by Gene Ontology analysis in U87-EVs-treated mNPCs revealed. **D** Top 10 biology processes of downregulated proteins in U87-EVs-treated mNPCs revealed by Gene Ontology analysis. **E** Top 10 signaling pathways of upregulated proteins in U87-EVs-treated mNPCs revealed by KEGG analysis. **F** Top 10 signaling pathways of downregulated proteins in U87-EVs-treated mNPCs revealed by KEGG analysis. **G** Western blotting confirmation of upregulated PI3K-Akt-mTOR pathway proteins in U87-EVs-treated mNPCs. Results are presented as the mean ± SD of three independent experiments. *p < 0.05, **p < 0.01, ***p < 0.001 and ****p < 0.0001, in comparison with 0 h

To determine the molecular contents that may be responsible for increased proliferation and migration of glioblastoma-EVs-treated mNPCs, we performed proteomic profiling of glioblastoma-EVs-treated mNPCs. Since U87-EVs, A172-EVs, and LN229-EVs exhibited similar influences on mNPCs’ regulation, we only used U87-EVs for following mechanism identification and confirmation studies. Proteomic analysis identified 5703 proteins in mNPCs with/without EVs treatment (Additional file 2: Table S1). Among them, there were 187 upregulated and 141 downregulated proteins in U87-EVs-treated mNPCs versus control mNPCs (Fig. 5A, Additional file 1: Fig. S2, Additional file 3: Table S2). These differentially expressed proteins were majorly located in cytoplasm (31.91%), nucleus (27.66%), and mitochandria (13.07%) (Fig. 5B). The expression levels of top up-regulated proteins (SMPD3, HIST1H2AK, and PUS3) and top down-regulated proteins (PEG3 and CNBP) revealed by proteomic analysis in U87-EVs-treated mNPCs were validated by western blotting (Additional file 1: Fig. S3). Importantly, the top up-regulated proteins (SMPD3, HIST1H2AK, and PUS3) were also up-regulated in A172-EVs-treated mNPCs and LN229-EVs-treated mNPCs, while the top down-regulated proteins (PEG3 and CNBP) were also down-regulated in A172-EVs-treated mNPCs and LN229-EVs-treated mNPCs (Additional file 1: Fig. S3). These results suggested that the regulatory profiles in U87-EVs-, A172-EVs-, and LN229-EVs-treated mNPCs were very similar.

Gene Ontology analysis revealed that the upregulated proteins were mainly involved in cancer-related biological processes in U87-EVs-treated mNPCs, such as positive regulation of cell cycle arrest, mRNA catabolic process, and positive regulation of cell proliferation, while the downregulated proteins were associated with RNA processing, transcription, and translation (Fig. 5C, D). KEGG analysis confirmed that the upregulated proteins in mNPCs treated by U87-EVs were strongly linked with cancer-related signaling pathways, such as Thyroid hormone signaling, AMPK signaling, and metabolic pathways (Fig. 5E). In contrast, the downregulated proteins were involved in pathways that induce cell stress and neurodegeneration including Huntington’s disease, Parkinson’s disease, and Oxidative phosphorylation (Fig. 5F).

To corroborate the proteomic analyses results above, we applied the proteins with 2 folds changes (20 upregulated proteins and 2 downregulated proteins) to GO and KEGG analyases. GO analysis identified 7 significantly enriched biological processes and the first one is protein phosphorylation. These results (Additional file 1: Fig. S4), together with GO and KEGG analysis of all differentially-expressed proteins (Fig. 5C, E), indicate the importance to study the effects of glioblastoma-derived EVs on the phosphorylation of PI3K-Akt components. Importantly, among the altered cell signaling pathways in U87-EVs-treated mNPCs (Fig. 5E), both thyroid hormone signaling pathway and AMPK signaling pathway are mediated by downstream PI3K-Akt signaling pathway. Interestingly, by analyzing the proteome data from published article from Haraszti et. al. [22], we confirmed that proteins participating in PI3K-Akt pathway were significantly enriched in U87-EVs (Additional file 1: Fig. S5) and specifically sorted into U87-EVs (Additional file 1: Fig. S6). To verify the results obtained from proteomic analyses, we next performed western blotting to examine the activities of PI3K-Akt pathway in U87-EVs-treated mNPCs (Fig. 5G). Indeed, we observed upregulation of PI3K-Akt signaling pathway molecules, including phosphorylated PI3K, Akt, and mTOR, post glioblastoma-derived EV treatment in an approximately time-dependent manner, indicating the induction of PI3K-Akt-mTOR signaling in mNPCs by U87-EVs.

### Glioblastoma-derived EVs increased the proliferation and migration of mNPCs through upregulating PI3K-Akt signaling

**Fig. 6:**
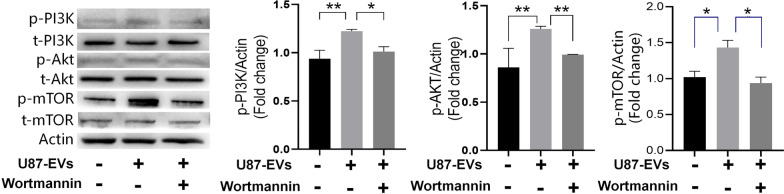
Wortamnnin lowered upregulated PI3K-Akt-mTOR pathway proteins in glioblastoma-derived EVs-treated mNPCs. Western blotting analysis revealed that Wortamannin treatment lowered the protein levels of p-PI3K, p-Akt, and mTOR. Results are presented as the mean ± SD of three independent experiments. *p < 0.05, **p < 0.01

To verify whether the elevated activities of PI3K-Akt signaling is responsible for enhanced mNPCs proliferation and migration after being treated by U87-EVs, we used 2.5 μM Wortmannin to block PI3K-Akt signaling in U87-EVs-treated mNPCs. As expected, Wortmannin treatment significantly inhibited the phosphorylation of PI3K, Akt, and mTOR (Fig. 6). Using western blotting, we showed that the expressions of phospho-PI3K (p-PI3K), p-Akt, and p-mTOR were significantly elevated by U87-EVs, A172-EVs, and LN229-EVs treatment in mNPCs. Wormannin treatment significantly reduced glioblastoma-derived EVs-induced PI3K-Akt upregulation, as demonstrated by reduced expressions of p-PI3K, p-Akt, and p-mTOR after Wortmannin treatment (Fig. 6).

**Fig. 7:**
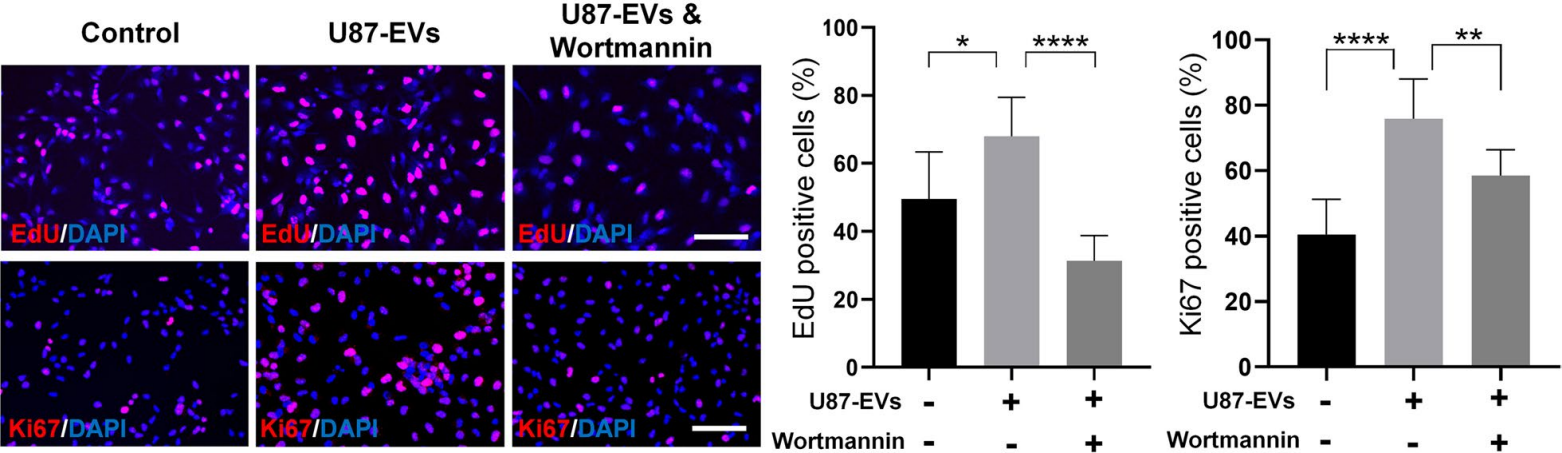
Inhibiting PI3K-Akt pathway in glioblastoma-derived EVs-treated mNPCs reversed elevated NPC proliferation. Wortamnnin significantly reversed U87-EVs-elevated proportion of EdU-positive cells in U87-EVs-treated mNPCs. Wortamnnin significantly reversed U87-EVs-elevated proportion of Ki67-positive cells in U87 EVs-treated mNPCs. Results are presented as the mean ± SD of three independent experiments. *p < 0.05, **p < 0.01, and ****p < 0.0001. Scale bar, 100 μm

**Fig. 8:**
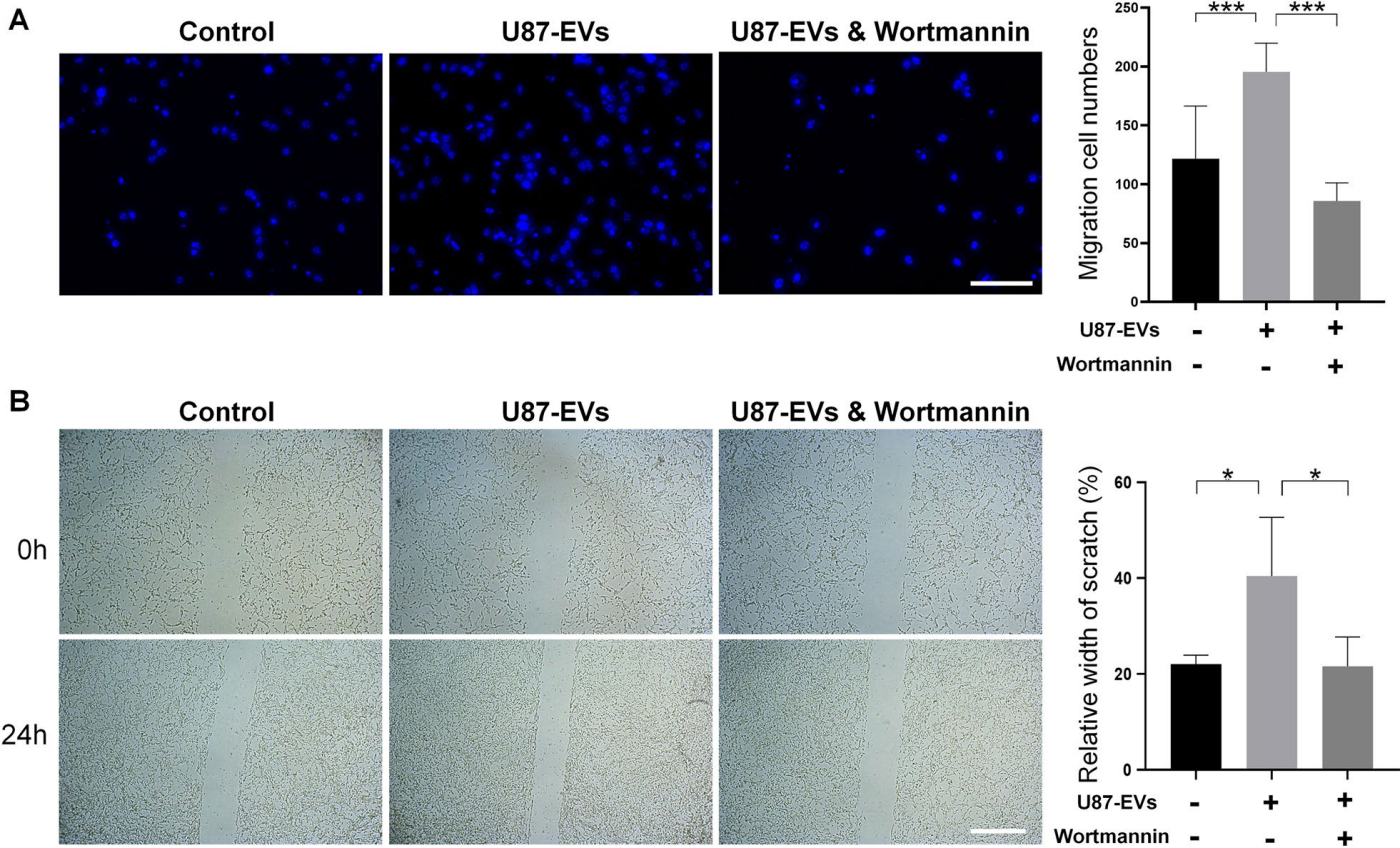
Inhibiting PI3K-Akt pathway in glioblastoma-derived EVs-treated mNPCs reversed elevated NPC migration. **A** Wortamnnin significantly reversed U87-EVs-elevated migration cell numbers in U87-EVs-treated mNPCs in the transwell assay. **B** Wortamnnin significantly reversed U87-EVs-elevated relative width of scratch in U87-EVs-treated mNPCs in wound healing assay. Results are presented as the mean ± SD of three independent experiments. *p < 0.05, ***p < 0.001. Scale bar, 100 μm (**A**) and 200 μm (**B**)

Importantly, both EdU incorporation assay and Ki67 immunocytochemistry revealed that the suppression of PI3K-Akt pathway by Wortmannin reversed the enhanced proliferation of U87-EVs-treated mNPCs (Fig. 7). Likewise, both transwell (Fig. 8A) and wound healing assay (Fig. 8B) showed that Wortmannin treatment significantly lowered the increased migration capacities of U87-EVs-treated mNPCs. These results showed that inhibiting PI3K-Akt pathway were able to abrogate the positive effects of glioblastoma-derived EVs on proliferation and migration of mNPCs, indicating the activation of PI3K-Akt pathway as a key intracellular mechanisms in the phenotype transtion of mNPCs that induced by TME.

## Discussion

Glioblastoma is known to affect almost all types of cells in the TME and recruit nonneoplastic cells to support tumor growth, subsistence, and encroachment into nontumor regions [8]. Glioblastoma cells stimulate angiogenesis and consolidate existing vasculature by secreting regulatory growth factors like vascular endothelial growth factor (VEGF) or hepatoma-derived growth factor (HDGF) [23, 24]. Glioblastoma suppresses both innate and adaptive immune cell functions: microglia are disarmed and lose their abilities to recognize foreign/toxic elements [25]; T cells are suppressed with a complex array of immune regulatory mechanisms to allow neoplastic cells escape the immune checkpoints [8, 26–28]. Peritumoral astrocytes’ transcriptome and secretome are altered by glioma resection to promote cellular proliferation and migration [9]. More importantly, glioblastoma cells induce NPCs to migrate toward the site of tumor [11] and exhibit transformed phenotypes [29]. In our study, we confirmed the positive effects of glioblastoma cells on proliferation and migration of mNPCs, clearly presenting the influence of TME on normal cells.

Cellular communication between tumor cells and surrounding cells plays a key role in this glioblastoma “hijacking” of the brain. Soluble factors secreted by tumor cells, like transforming growth factor-β (TGFβ), IL-6, platelet-derived growth factor (PDGF), epidermal growth factor (EGF), VEGF, and stromal cell-derived factor 1 (SDF1; also known as CXCL12), serve as important signaling molecules as they bind to receptors located on target cells [11]. Apart from soluble factors, tumor cells utilize other forms of communication to transfer non-secreted factors like genetic materials, transcription factors, and even organelles like mitochondria or nuclei via gap junctions, EVs, or nanotubes [8, 30, 31]. These different routes of interactions between tumor cells and nonneoplastic cells in the TME facilitate glioblastoma to create a flexible environment that adapts to challenges like resection, chemo-, or radiotherapy. Among aforementioned factors, EVs have emerged as a key communicator in the TME to transfer both soluble and non-soluble contents from donor cells to recipient cells. In glioblastoma, EVs derived from donor tumor cells transfer soluble factors like cytokines and chemokines, as well as non-soluble molecules like transcription factors, RNA, DNA, lipids, and metabolites, to normal cells and subsequently change the phenotypes of the latter [32, 33]. In this study, we show that EVs mediate the influences of glioblastoma on proliferation and migration of NPCs. Our study provides new evidences for the intercellular communication between glioblastoma cells and NPCs, which further substantiates the components of TME and extends the interaction network of these components.

By delivering bioactive cargos into recipient cells, EVs can influence various intracellular signaling pathways. In our study, we identified that PI3K-Akt pathway as the key one that mediates the positive effects of EVs on proliferation and migration of mNPCs. PI3K-Akt signaling is a hyperactivated pathway in cancer cells that regulates diverse cellular functions including survival, proliferation, migration, and metabolism [34, 35]. More than 60% of glioblastoma cases have been linked with at least one mutated or dysregulated PI3K-Akt-mTOR pathway proteins [36–38]. Mutations of *PIK3CA*, the gene encoding the p110α subunit of PI3K, or mutations of *PIK3R1*, the gene encoding the p85 regulatory subunit of PI3K, have been demonstrated in approximately 15% of patients with glioblastoma [38–40]. Loss of function, chromosomal abnormalities, or epigenetic gene silencing of PTEN, the negative regulator of PI3K-Akt pathway, have been found in approximately 40% of glioblastoma cases and have been linked with poor prognosis [41]. PI3K-Akt signaling partly regulates hypoxia-inducible-factor (HIF) stabilization, which promotes glioblastoma division [42]. The PI3K-Akt pathway-induced HIF activation also modulates Warburg effect, the most widely-accepted metabolic shift that cancer cells heavily depend on for additional energy supply [34, 43]. More importantly, activation of PI3K-Akt pathway in mNPCs directly induced glioblastoma formation in vivo, suggesting the direct link of PI3K-Akt pathway to glioblastoma-induced NPC phenotype change [44]. Furthermore, studies show that activation of PI3K-Akt-mTOR enhanced tumor cell tolerance to chemotherapy [45]. To date, to overcome the commonly occurred radio- or chemo-resistance, immunotherapy is introduced in combination with surgery, chemo, and radiotherapy in glioma treatment. Multiple strategies of glioblastoma immunotherapy are via PI3K-Akt signaling inhibition [46, 47]. These therapies prove effective in animal models, however, almost fail to make any difference in clinic given the high heterogeneity of cellular and molecular components in glioblastoma [8, 48]. Blocking EVs release from glioma tumor cells might be a promising strategy to inhibit the activation of PI3K-Akt pathway from the source in combination with other forms of therapy, which, needs to be further studied in future research.

Interestingly, our results did reveal that components of MEK-ERK pathway were also significantly upregulated in a time-dependent manner (Additional file 1: Fig. S7), demonstrating heightened expressions of cancerous shift-related signaling molecules other than PI3K-Akt pathway components in U87 EVs-treated mNPCs. MEK-ERK pathway is strongly associated with poor prognosis and glioblastoma patient survival [49]. In glioblastoma, MEK-ERK pathway has been reported to promote angiogenesis once activated by VEGF under hypoxia [49], to enhance drug resistance [50], and to maintain high glutamine metabolism [51]. Similar to PI3K-Akt pathway, MEK-ERK signaling has also been considered as a therapeutic target of glioblastoma. Ethyl pyruvate [52], trametinib [53], amentoflavone [54], hyperforin [55], berberine [56], imipramine [57], and protein neddylation inhibitor MLN4924 [58] inhibit glioblastoma cells migration and invasion via suppressing MEK-ERK pathway. Among them, several drugs have been applied in clinical trials such as trametinib (ClinicalTrials.gov Identifier: NCT03363217) [53]. In our study, the treatment of Wortmannin also significantly inhibited the activation of MEK-ERK pathway that is induced by U87-EVs (Additional file 1: Fig. S8), suggesting that MEK-ERK pathway may act as a downstream factor of PI3K-Akt pathway and both pathways may work together in mediating the tumorigenic effects of glioblastoma-derived EVs.

Apart from proliferation and migration, it is also important to know that whether glioblastoma-derived EVs affect mNPCs’ other cellular behaviors. We examined the differentiation of mNPCs after being co-culture with glioblastoma-derived EVs for 3 days in differentiation conditions. Surprisingly, all three glioblastoma-derived EVs exhibited positive roles in the differentiation of mNPCs, ascertained by both immunocytochemistry (Additional file 1: Fig. S9A, B) and qRT-PCR (Additional file 1: Fig. S9C) analyses. The underlying mechanism is unclear at current stage and will be investigated in our future studies. We also examined that whether the secretome profile of mNPCs altered with glioblastoma-derived EVs treatment. No significant alteration on the concentration of TNF-α and IL-1β in the culture medium of mNPCs with/without glioblastoma-derived EVs treatment was detected using ELISA (Additional file 1: Fig. S10). Given the fact that glioblastoma-derived EVs significantly alter the cellular behaviors of mNPCs, we are interested in that whether glioblastoma-derived EVs promote the epithelial to mesenchymal transition (EMT) of mNPCs [59]. Western blotting analysis revealed no significant change of the expression levels of EMT regulators including Snail and Slug between EVs-treated mNPCs and controls (Additional file 1: Fig. S11). More importantly, EVs treatment elevated the expression of E-cadherin in mNPCs, suggesting a positive effect of glioblastoma-derived EVs on the maintenance of certain epithelial features. Thus, our results indicated that glioblastoma-derived EVs did not confer a EMT phenotype to mNPCs.

Except for EMT, there comes an important question that whether or not glioblastoma cell derived-EVs confer a mesenchymal-like glioma stem cell (GSC)-like phenotype to mNPCs [60]. Presenting stem cell properties like self-renewal and multi-lineage differentiation potentials, GSCs are resistant to radiotherapy and have the potential to induce angiogenesis, metastasis and modulate therapeutic responses [61]. Compared with non-stem tumor cells, GSCs present a heightened DNA repair capacity and recover rapidly from conventional therapeutic stress [60, 62]. However so far, due to that a subpopulation of GSCs shares the same set of biomarkers with normal NPCs [63, 64], there remains in lack of direct evidence to precisely define the transition of mNPCs to GSCs. Therefore, it is difficult to prove that glioblastoma-derived EVs alter normal NPCs to acquire GSC phenotype, although our results indicate significant upregulation of cancer metabolic signaling molecules in EV-treated mNPCs and that these mNPCs present elevated proliferation and migration capacity. In future studies, more comprehensive investigations with new approaches and indexes should be developed to distinguish GSCs from NPCs and the effects of glioblastoma-derived EVs on mNPC phenotype transition should be further validated.

## Conclusions

Taken together, our study demonstrated glioblastoma-derived EVs promoted proliferation and migration of glioblastoma cells and mNPCs, suggesting the alteration of mNPCs’ phenotype via glioblastoma-derived EVs treatment. Furthermore, we identified key downstream factor of glioblastoma-derived EVs, PI3K-Akt pathway, as elevated proliferation and migration of mNPCs post EVs treatment could be reversed by PI3K inhibitor Wortamnnin. These findings expanded our current knowledge of tumor-nontumor interactions in the glioblastoma microenvironment and provided a new direction to overcome the current challenges (such as frequent recurrence) of glioblastoma treatment.

## Supplementary Information

**Additional file 1: Figure S1.** Characterization of EVs derived from U87, A172, and LN229 cells. (**A**–**C**) NTA characterization of U87-EVs (**A**), A172-EVs (**B**), and LN229-EVs (**C**). (**D**–**F**) Western blotting characterization of U87-EVs (**D**), A172-EVs (**E**), and LN229-EVs (**F**). Figure **S**2. Heatmap of differentially expressed proteins in U87-EVs-treated mNPCs versus control mNPCs. Figure **S**3. The validation of proteomic analysis through western blotting. (A) The expression levels of top up-regulated proteins (SMPD3, HIST1H2AK, and PUS3) and the top down-regulated proteins (PEG3 and CNBP) in glioblastoma cell-derived EVs-treated mNPCs, identified by the proteomic analysis, were determined by western blotting. Data were represented as mean ± s.d. from three independent experiments. *p < 0.05, **p < 0.01, and ***p < 0.001. **Figure S4.** GO analysis for 22 differentially expressed proteins with 2 folds changes between U87-EVs-treated mNPCs and control cells. Figure **S**5. KEGG analysis for top 100 abundantly expressed proteins in U87-EVs. **Figure S6.** Proteomic analysis of U87-EVs versus U87 cells. (**A**) Heatmap and hierarchical clustering of differentially expressed proteins in U87-EVs versus U87 cells. (**B**) Top 10 biology processes of upregulated proteins revealed by Gene Ontology analysis. (**C**) Top 10 signaling pathways of upregulated proteins revealed by KEGG analysis. **Figure S7.** Western blot confirmation of upregulated MEK-ERK signaling pathway proteins in U87-EVs-treated mNPCs. p–c-Raf, p-MEK, and p-ERK were upregulated in U87-EVs-treated mNPCs compared with control mNPCs in a time-dependent manner. Data were represented as mean ± s.d. from three independent experiments. *p < 0.05, **p < 0.01, and ***p < 0.001. **Figure S8.** Wortamnnin lowered upregulated MEK-ERK pathway proteins in U87-EVs-treated mNPCs. Western blot analysis revealed that PI3K inhibitor Wortamannin treatment lowered the protein levels of p–c-Raf, p-MEK, and p-ERK. Data were represented as mean ± s.d. from three independent experiments. *p < 0.05. **Figure S9.** Glioblastoma cell-derived EVs promote the differentiation of mNPCs. (**B**) U87-EVs, A172-EVs, and LN229-EVs treatments all significantly increased the proportions of EdU positive or Ki67 positive mNPCs. (**C**) U87-EVs, A172-EVs, and LN229-EVs treatments all significantly increased the expression levels of transcripts corresponding to *βIII-tubulin* and *GFAP* in mNPCs. Data were represented as mean ± s.d. from three independent experiments. *p < 0.05, **p < 0.01, ***p < 0.001, and ****p < 0.0001. Scale bar, 50 μm (**A**). **Figure S10.** Glioblastoma cell-derived EVs do not alter the secretome of mNPCs. (**B**) U87-EVs, A172-EVs, and LN229-EVs treatment all significantly increased the proportions of EdU positive or Ki67 positive mNPCs. (**C**) U87-EVs, A172-EVs, and LN229-EVs treatment all significantly increased the expression levels of transcripts corresponding to *βIII-tubulin* and *GFAP* in mNPCs. Data were represented as mean ± s.d. from three independent experiments. **Figure S11.** Glioblastoma cell-derived EVs have no effects on the expression of EMT-related proteins in NPCs. Western blot analysis revealed that glioblastoma cell-derived EVs did not alter the protein levels of N-CAD, E-CAD, Snail, and Slug

**Additional file 2: Table S1.** All detected proteins

**Additional file 3: Table S2.** Differentially expressed proteins

## Data Availability

The datasets generated for this study are available on reasonable request to the corresponding authors.

## Abbreviations

DLS: Dynamic light scattering
EGF: Epidermal growth factor
EMT: Epithelial to mesenchymal transition
EVs: Extracellular vesicles
GSCs: Glioma stem cells
HDGF: Hepatoma-derived growth factor
HIF: Hypoxia-inducible-factor
IL: Interleukin
mNPCs: Mouse neural progenitor cells
NPCs: Neural progenitor cells
NTA: Nanoparticle trafficking
PDGF: Platelet-derived growth factor
PFA: Paraformaldehyde
TEM: Transmission electron microscopy
TGFβ: Transforming growth factor-β
TME: Tumor microenvironment
TNF-α: Tumor necrosis factor alpha
VEGF: Vascular endothelial growth factor
