# Supplementary material for: Extracellular vesicles derived from glioblastoma promote proliferation and migration of neural progenitor cells via PI3K-Akt pathway
Source: Cell Commun Signal. 2022 Jan 12;20:7. doi: 10.1186/s12964-021-00760-9 (PMC8756733; doi:10.1186/s12964-021-00760-9)
Supplement: Supplementary file 2 — Additional file 1: Figure S1. Characterization of EVs derived from U87, A172, and LN229 cells. (A–C) NTA characterization of U87-EVs (A), A172-EVs (B), and LN229-EVs (C). (D–F) Western blotting characterization of U87-EVs (D), A172-EVs (E), and LN229-EVs (F). Figure S2. Heatmap of differentially expressed proteins in U87-EVs-treated mNPCs versus control mNPCs. Figure S3. The validation of proteomic analysis through western blotting. (A) The expression levels of top up-regulated proteins (SMPD3, HIST1H2AK, and PUS3) and the top down-regulated proteins (PEG3 and CNBP) in glioblastoma cell-derived EVs-treated mNPCs, identified by the proteomic analysis, were determined by western blotting. Data were represented as mean ± s.d. from three independent experiments. *p < 0.05, **p < 0.01, and ***p < 0.001. Figure S4. GO analysis for 22 differentially expressed proteins with 2 folds changes between U87-EVs-treated mNPCs and control cells. Figure S5. KEGG analysis for top 100 abundantly expressed proteins in U87-EVs. Figure S6. Proteomic analysis of U87-EVs versus U87 cells. (A) Heatmap and hierarchical clustering of differentially expressed proteins in U87-EVs versus U87 cells. (B) Top 10 biology processes of upregulated proteins revealed by Gene Ontology analysis. (C) Top 10 signaling pathways of upregulated proteins revealed by KEGG analysis. Figure S7. Western blot confirmation of upregulated MEK-ERK signaling pathway proteins in U87-EVs-treated mNPCs. p–c-Raf, p-MEK, and p-ERK were upregulated in U87-EVs-treated mNPCs compared with control mNPCs in a time-dependent manner. Data were represented as mean ± s.d. from three independent experiments. *p < 0.05, **p < 0.01, and ***p < 0.001. Figure S8. Wortamnnin lowered upregulated MEK-ERK pathway proteins in U87-EVs-treated mNPCs. Western blot analysis revealed that PI3K inhibitor Wortamannin treatment lowered the protein levels of p–c-Raf, p-MEK, and p-ERK. Data were represented as mean ± s.d. from three independent experime [file 12964_2021_760_MOESM2_ESM.docx]

**Extracellular vesicles derived from glioblastoma promote proliferation and migration of neural progenitor cells via PI3K-Akt pathway**

**Jiabin Pan, Shiyang Sheng, Ling Ye, Xiaonan Xu, Yizhao Ma, Xuanran Feng, Lisha Qiu, Zhaohuan Fan, Yi Wang, Xiaohuan Xia, Jialin C. Zheng**

**Supplementary Materials**

Supplementary Figure 1

Supplementary Figure 2

Supplementary Figure 3

Supplementary Figure 4

Supplementary Figure 5

Supplementary Figure 6

Supplementary Figure 7

Supplementary Figure 8

Supplementary Figure 9

Supplementary Figure 10

Supplementary Figure 11


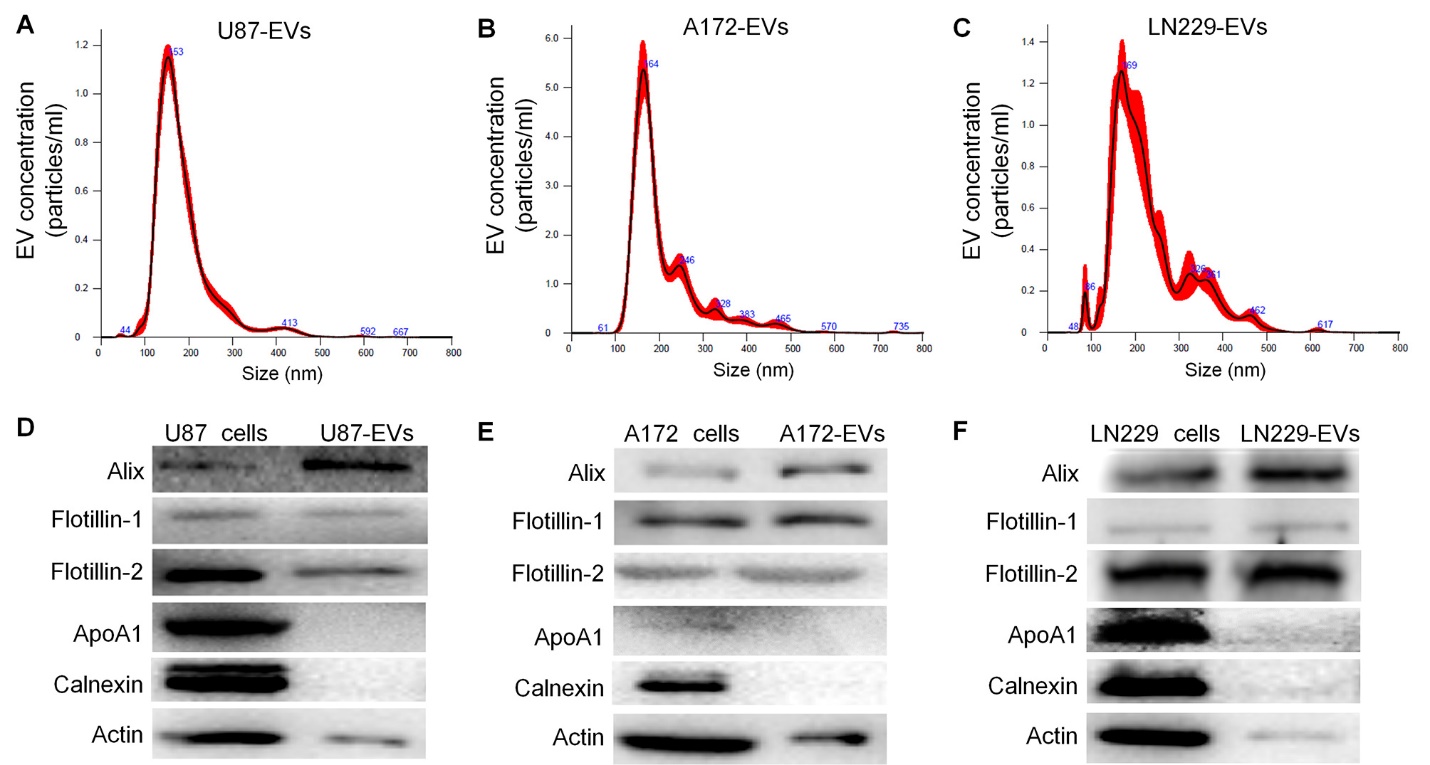


**Supplementary Figure 1. Characterization of EVs derived from U87, A172, and LN229 cells.**

**(A-C)** NTA characterization of U87-EVs (**A**), A172-EVs (**B**), and LN229-EVs (**C**). **(D-F)** Western blotting characterization of U87-EVs (**D**), A172-EVs (**E**), and LN229-EVs (**F**).

**
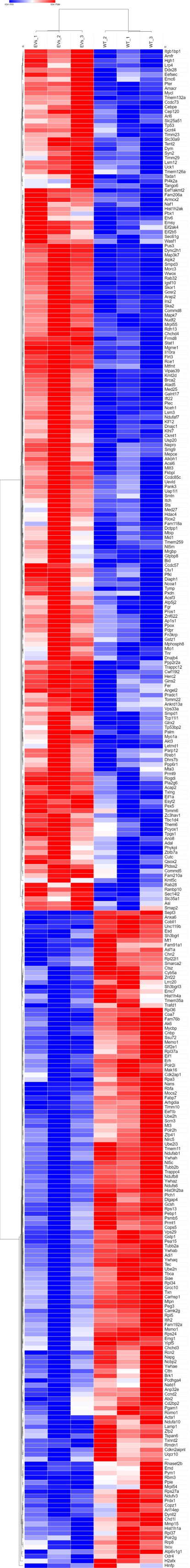
**

**
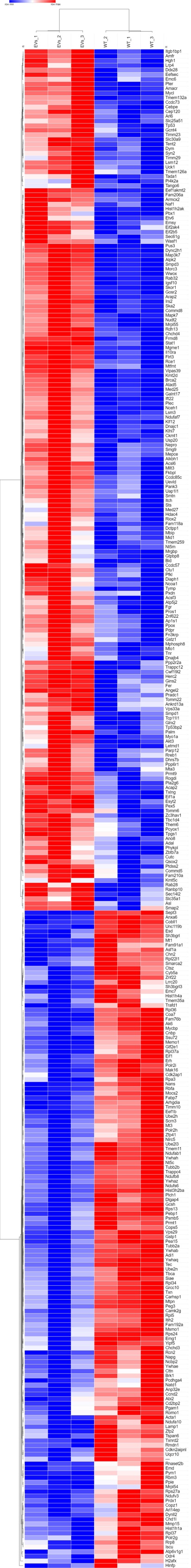
**

**Supplementary Figure 2**. **Heatmap of differentially expressed proteins in U87-EVs-treated mNPCs versus control mNPCs.**

**
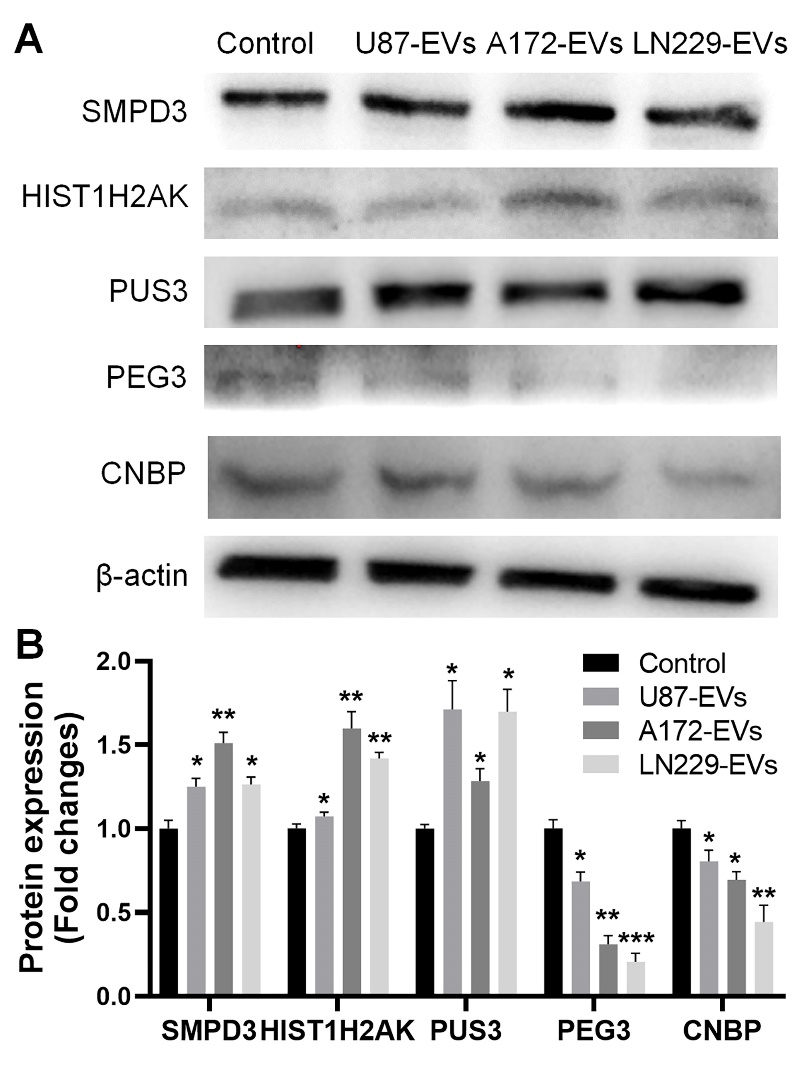
**

**Supplementary Figure 3**. **The validation of proteomic analysis through western blotting.**

(**A**) The expression levels of top up-regulated proteins (SMPD3, HIST1H2AK, and PUS3) and the top down-regulated proteins (PEG3 and CNBP) in glioblastoma cell-derived EVs-treated mNPCs, identified by the proteomic analysis, were determined by western blotting. Data were represented as mean ± s.d. from three independent experiments. **p*<0.05, ***p*<0.01, and ****p*<0.001.

**
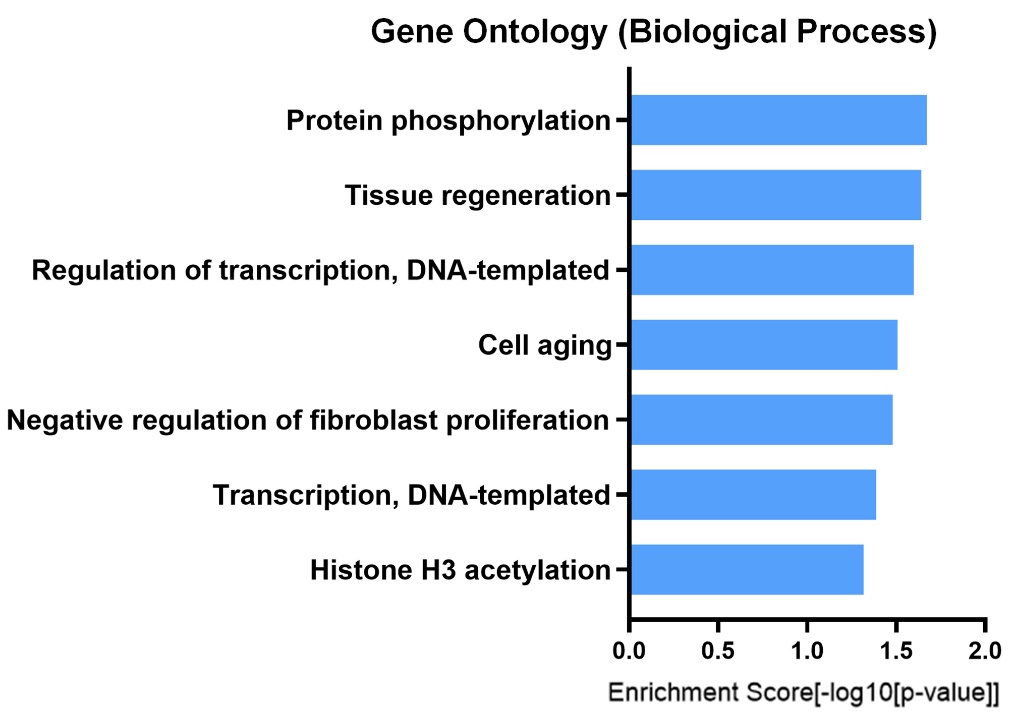
**

**Supplementary Figure 4. GO analysis for 22 differentially expressed proteins with 2 folds changes between U87-EVs-treated mNPCs and control cells.**

**
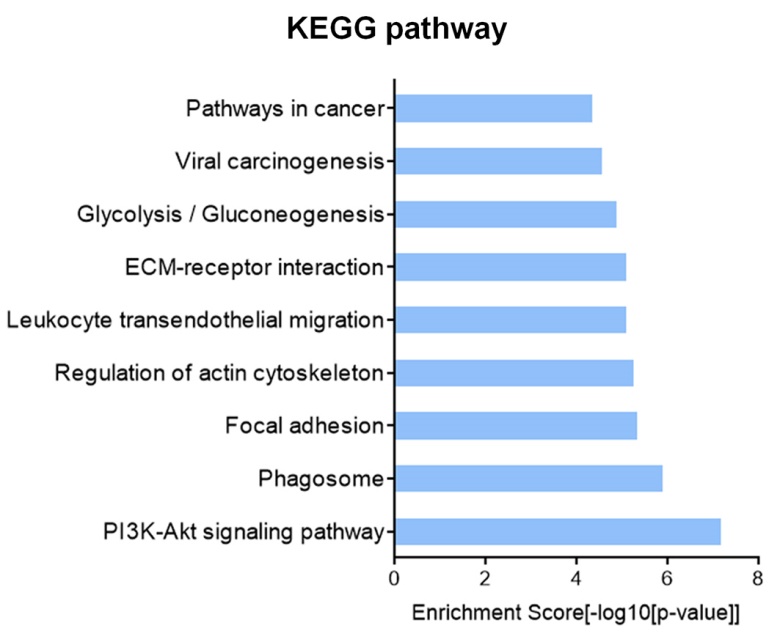
**

**Supplementary Figure 5. KEGG analysis for top 100 abundantly expressed proteins in U87-EVs.**

**
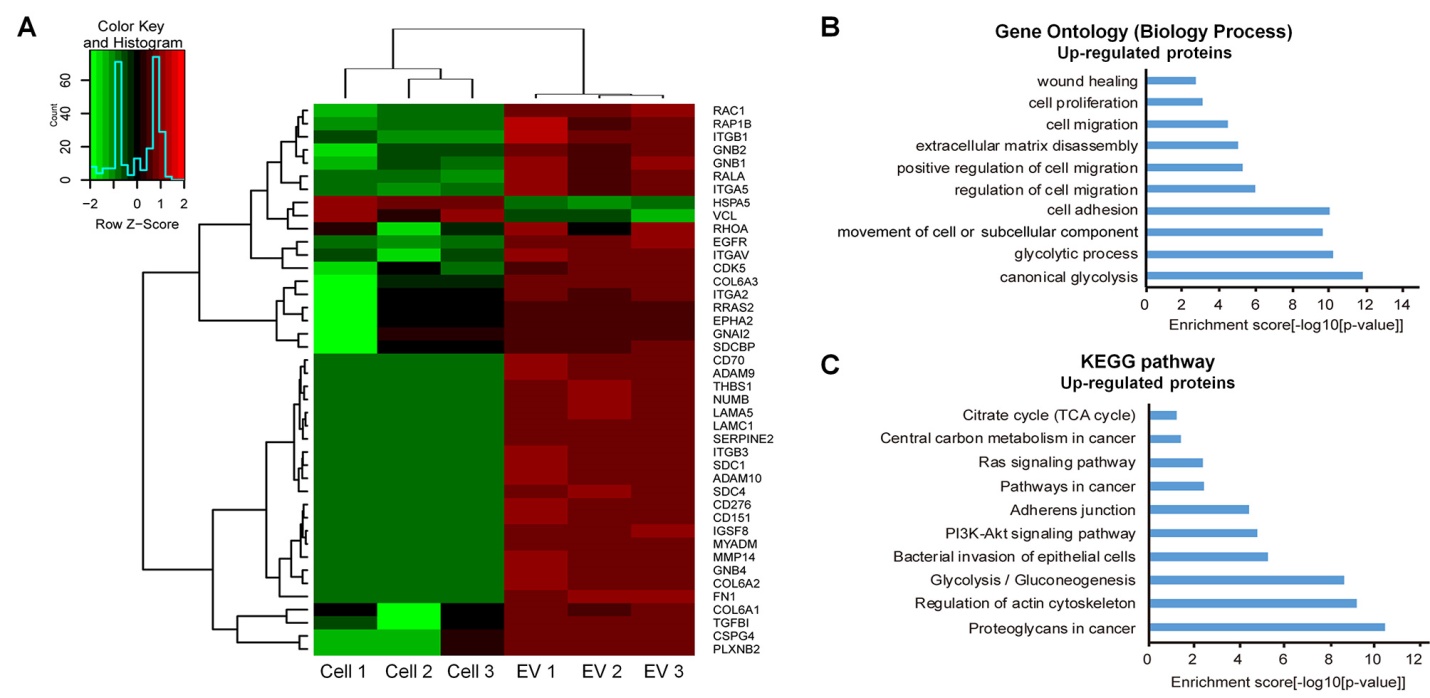
**

**Supplementary Figure 6**. **Proteomic analysis of U87-EVs versus U87 cells.**

**(A)** Heatmap and hierarchical clustering of differentially expressed proteins in U87-EVs versus U87 cells. (**B)** Top 10 biology processes of upregulated proteins revealed by Gene Ontology analysis. (**C)** Top 10 signaling pathways of upregulated proteins revealed by KEGG analysis.


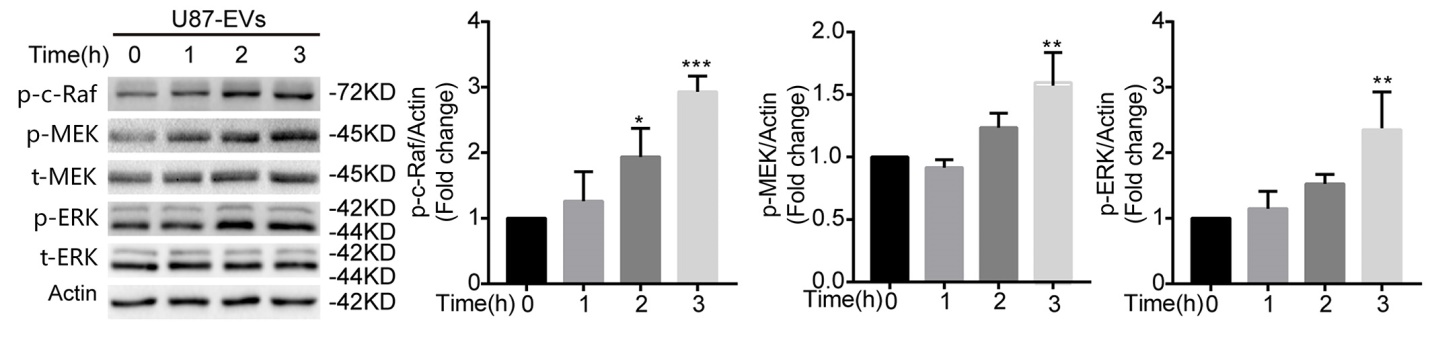


**Supplementary Figure 7**. **Western blot confirmation of upregulated ERK signaling pathway proteins in U87-EVs-treated mNPCs.**

p-c-Raf, p-MEK, and p-ERK were upregulated in U87-EVs-treated mNPCs compared with control mNPCs in a time-dependent manner. Data were represented as mean ± s.d. from three independent experiments. **p*<0.05, ***p*<0.01, and ****p*<0.001.


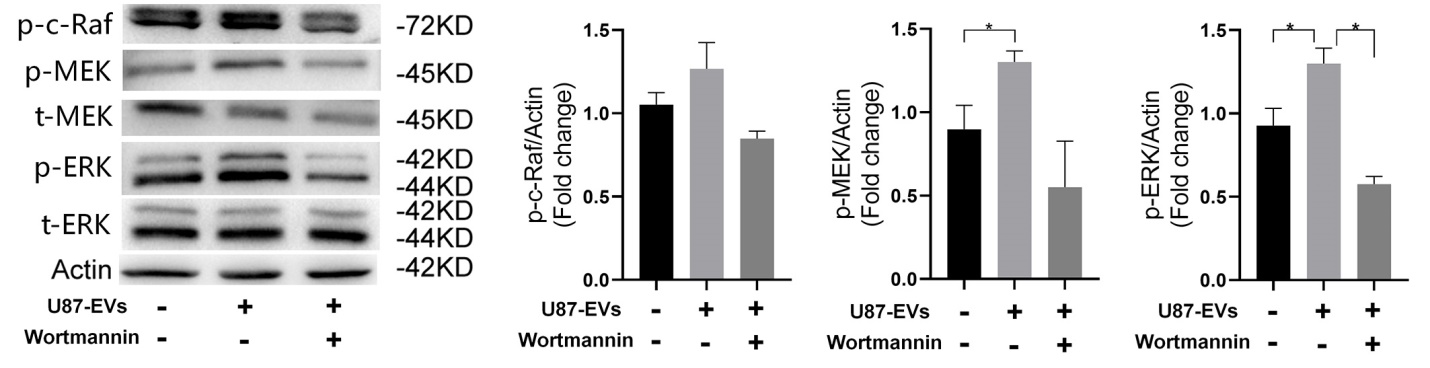


**Supplementary Figure 8**. **PI3K inhibitor Wortamnnin lowered upregulated ERK pathway proteins in U87-EVs-treated mNPCs.**

Western blot analysis revealed that PI3K inhibitor Wortamannin treatment lowered the protein levels of p-c-Raf, p-MEK, and p-ERK. Data were represented as mean ± s.d. from three independent experiments. **p*<0.05.


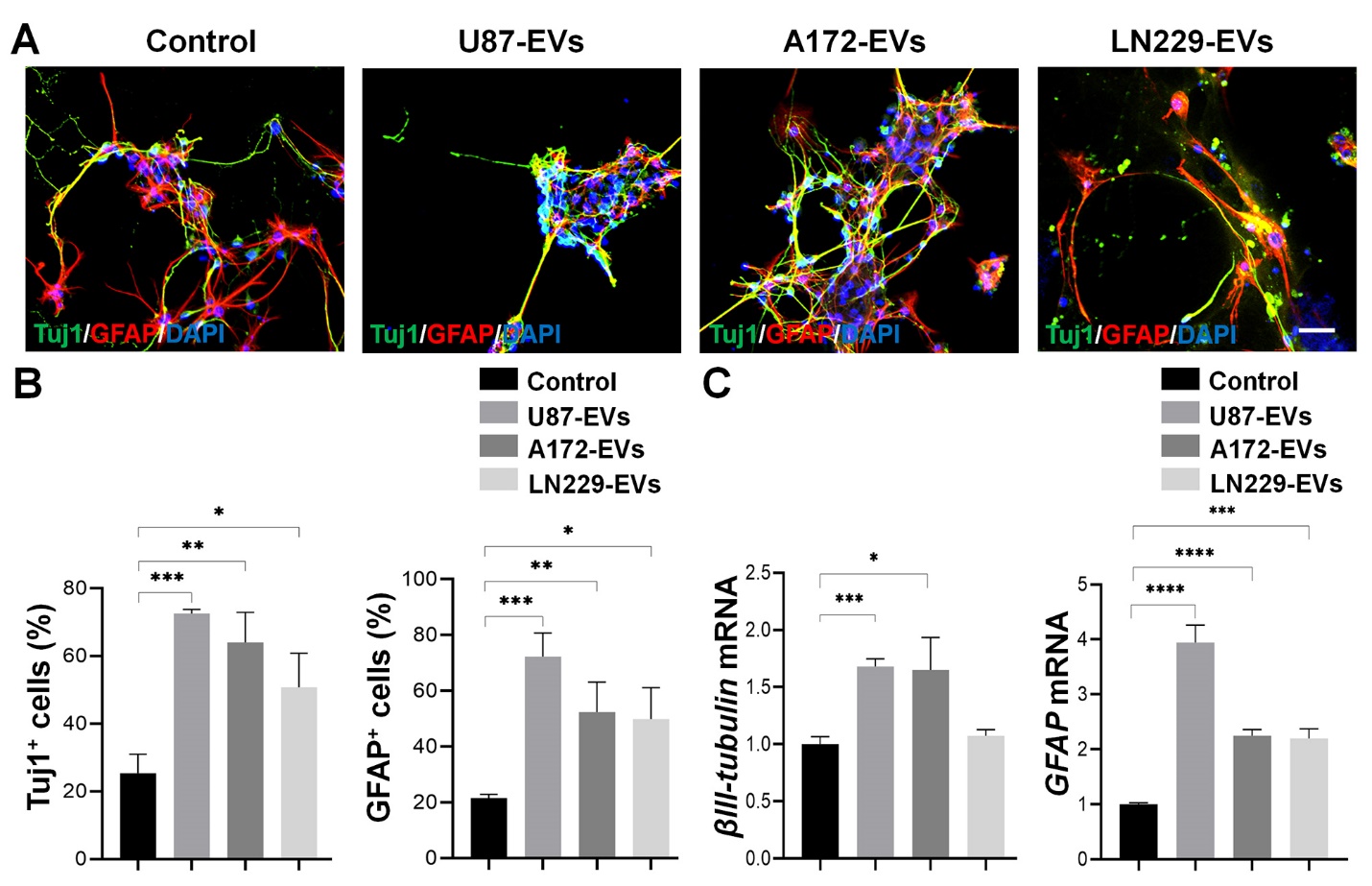


**Supplementary Fig 9. Glioblastoma cell-derived EVs promote the differentiation of mNPCs.**

**(B)** U87-EVs, A172-EVs, and LN229-EVs treatments all significantly increased the proportions of EdU positive or Ki67 positive mNPCs. **(C)** U87-EVs, A172-EVs, and LN229-EVs treatments all significantly increased the expression levels of transcripts corresponding to *βIII-tubulin* and *GFAP* in mNPCs. Data were represented as mean ± s.d. from three independent experiments. **p*<0.05, ***p*<0.01, ****p*<0.001, and *****p*<0.0001. Scale bar, 50 μm (**A**).


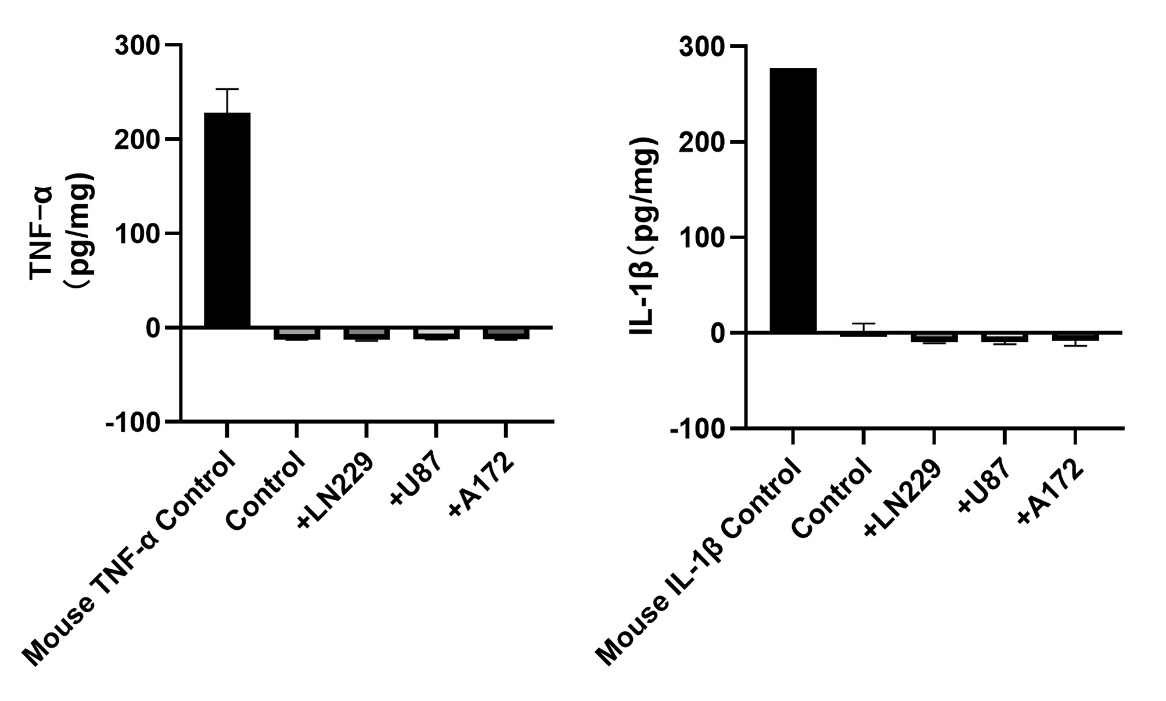


**Supplementary Fig 10. Glioblastoma cell-derived EVs do not alter the secretome of mNPCs.**

**(B)** U87-EVs, A172-EVs, and LN229-EVs treatment all significantly increased the proportions of EdU positive or Ki67 positive mNPCs. **(C)** U87-EVs, A172-EVs, and LN229-EVs treatment all significantly increased the expression levels of transcripts corresponding to *βIII-tubulin* and *GFAP* in mNPCs. Data were represented as mean ± s.d. from three independent experiments.


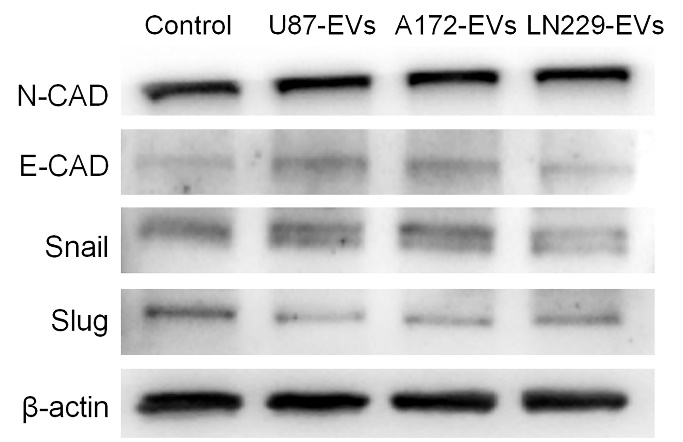


**Supplementary Figure 11**. **Glioblastoma cell-derived EVs have no effects on the expression of EMT-related proteins in NPCs.**

Western blot analysis revealed that glioblastoma cell-derived EVs did not alter the protein levels of N-CAD, E-CAD, Snail, and Slug.
